# Wheat genotypes selected for their high early daytime stomatal conductance under elevated nocturnal temperatures maintain high yield and biomass

**DOI:** 10.1093/aobpla/plae072

**Published:** 2024-12-25

**Authors:** R Suzuky Pinto, Jaime Garatuza-Payán, Erik H Murchie, Matthew P Reynolds, Enrico A Yepez

**Affiliations:** Departamento de Ciencias de Agua y Medio Ambiente, Instituto Tecnológico de Sonora, 5 de Febrero 818 Sur, Col. Centro, Cd. Obregón, Sonora, 85000, México; Departamento de Ciencias de Agua y Medio Ambiente, Instituto Tecnológico de Sonora, 5 de Febrero 818 Sur, Col. Centro, Cd. Obregón, Sonora, 85000, México; Division of Plant and Crop Sciences, School of Biosciences, University of Nottingham, Sutton Bonington, Leicestershire, LE12 5RD, United Kingdom; International Maize and Wheat Improvement Centre (CIMMYT), Carretera México-Veracruz Km 45, El Batán, Texcoco, 56237, Mexico; Departamento de Ciencias de Agua y Medio Ambiente, Instituto Tecnológico de Sonora, 5 de Febrero 818 Sur, Col. Centro, Cd. Obregón, Sonora, 85000, México

**Keywords:** circadian, transpiration, heat, climate change, wheat, *Triticum aestivum*, *Triticum durum*, physiological breeding

## Abstract

Global night-time temperatures are increasing and correlate with a decline in crop yield. Various aspects of nocturnal physiology in plants are understudied, one of which is the independent influence on daytime processes. Twelve elite wheat genotypes were field grown in plots with artificially increased night-time temperatures (+ 2°C). The stomatal conductance on the adaxial and abaxial leaf sides were measured during the morning and the night, and stomatal morphological traits were assessed during the same period of plant growth. To determine whether an increase in early daytime stomatal conductance provides a growth and/or yield advantage under high temperature, the biomass (Bm), grain number (Gn), thousand-grain weight (TGW), and grain yield (Yld) were measured. Genotypes exhibiting the highest early daytime stomatal conductance also showed higher Bm, Gn, and Yld. An increase of 19% in early daytime stomatal conductance led to a Yld increase of 86 and 65 gm^-2^, in heated and control conditions respectively, translating to 43 and 35% Yld enhancement. Irrespective of the environment, the adaxial leaf side showed the highest diurnal and nocturnal conductance, mirroring direct stomatal aperture (SA) measurements. The high night-time temperature treatment increased daytime stomatal conductance but reduced nocturnal conductance. SA varied with growing conditions; in the morning, plants under high night-time temperatures showed larger SA than those from the control independently of the leaf side, conversely, the opposite trend was observed in the night. Stomata on the adaxial leaf side showed higher density and larger size. Results from this study show that early daytime conductance increased productivity in hot-irrigated environments studied here. Previous studies showed that high pre-dawn conductance improves morning photosynthesis, and here we find that high nocturnal temperatures increased early morning conductance but reduced night-time conductance and this may be a factor that contributed to minimize Yld losses.

## Teaser

Nocturnal temperature is increasing faster than diurnal temperature but its effects on plant development are poorly understood. The stomata are the structures responsible for the release and capture of gases between the plant and the atmosphere which is why their characterization is crucial. In this paper we show differences in stomata density, size and ratio across a set of wheat genotypes. Differences also exist between both leaf sides and between growing conditions. Additionally, genotypes exhibiting the highest stomatal conductance during the first hours of the day were those grown under high nighttime temperature and still more interesting, these genotypes with increased early morning stomatal conductance showed up to 43% higher yield. We hypothesize an involvement of the circadian rhythm driving this favorable response.

## Introduction

High-temperature stress, especially during the reproductive stage is a major limiting factor that impacts the development and productivity of wheat (Cossani and Reynolds 2012; Bita and Gerats 2013; García et al. 2016). An understudied aspect is the impact of night-time temperatures which are increasing faster than daytime temperatures (Sillmann et al. 2013; IPCC 2021). In recent studies, grain yield Yld has been reported to decrease by 1.9% for every 1°C increase in nocturnal temperatures (minimum temperatures) of spring wheat but the spectrum is wide with reports of up to 10% for every 1°C increase (Lobell and Ortiz-Monasterio 2007; McAusland et al. 2023) and 12.9% in winter wheat (Hein et al. 2019). With increasingly hot climate scenarios and the occurrence of more frequent and severe heat waves, these penalties are expected to get worse in the coming years (Lobell et al. 2005; Rezaei et al. 2023). Minimum temperatures can have a significant impact on plant performance and reduce Yld by limiting the number of grains, accelerating plant senescence, raising photoassimilates consumption, and reducing gas exchange rates and levels of carbon assimilates available to fill grains (Mohammed and Tarpley 2009; Xu et al. 2021; Parveen et al. 2022).

Gas exchange between the plant and the atmosphere is partly determined by the physical leaf characteristics including the stomatal morphology and leaf structure which influence paths for CO_2_ and H_2_O transfer (Lawson and Matthews 2020; Mizokami et al. 2022; Wall et al. 2022). Morphological diversity of stomata is widely documented across species, and within wheat cultivars, however, its relevance for wheat physiology and crop improvement is not clear. Wheat possesses ‘dumb-bell’ shaped stomata typical of grasses, the guard cells of which have been observed to be smaller and faster moving than dicotyledonous types (Nunes et al. 2020). The number and size of stomata seem to be a strongly genetically determined trait with species dependency and the stomatal number per mm^2^ has been reported to depend on the ploidy level (Wilson et al. 2021; Haworth et al. 2023). However, there is evidence of further phenotypic plasticity raising the question of whether this could confer an advantage under suboptimal growing conditions, enabling the plant to develop the most convenient stomata size and densities to deal with environments of limited water availability, intensive radiation, high temperature or variation in CO_2_ levels (Mansfield et al. 1990; Schneider 2022; Haworth et al. 2023). This is supported by data showing that a substantial reduction in wheat stomatal density (SD) leads to greater water use efficiency (Dunn et al. 2019). Wheat leaves are amphistomatic, which means that stomata can be found on both, the adaxial and abaxial leaf sides; the latter is thought to facilitate gas diffusion through the mesophyll by shortening the pathway for CO_2_ transport from the atmosphere to the chloroplasts (Mott et al. 1982; Drake et al. 2019). However, significant differences exist between leaf sides, the adaxial contains the highest number of stomata, and authors report higher rates of gas exchange compared to the abaxial leaf side rate (Bi et al. 2017; Wall et al. 2022). Nonetheless, gas exchange depends not only on the stomata size, location, and density, but also on the stomata ability to open and close rapidly in response to environmental cues. Stomatal behavior has been linked to the shape of guard cells, the morphology and size of the stomata, and the alignment of subsidiary cells. Smaller numerous stomata are thought to facilitate gas exchange by shortening path length and providing more rapid stomata opening and closing (Franks and Farquhar 2007). A higher speed of stomatal opening in response to light can potentially maximize carbon gains; important when considering the varying light conditions derived from leaf angle and leaf position in the canopy, or due to temporal environmental fluctuation in photon flux density (PPFD). Slow stomata closing when PPFD decreases can result in unnecessary water losses while slow stomata opening when PPFD increases will limit carbon gains (Lawson and Vialet-Chabrand 2019; Long et al. 2022). The speed of stomata movements depends on the mechanical interaction between the guard and epidermal cells of the stomata and on the evaporative demand on the environments, this is the vapour pressure deficit (VPD) (Pichaco et al. 2024). There is no consensus about the association between CO_2_ assimilation/H_2_O losses and stomata morphology. For example, (Wilson et al. 2021) found that wheat species with higher stomatal densities do not correspond to those with the highest stomatal conductance, but it is now widely accepted that stomata remain partially open during the night-time resulting in nocturnal water losses up to 55% of daytime losses (Schoppach et al. 2014). Molecular and gene signalling associated with the CCA1 clock gene have been shown to induce stomata closure in *Arabidopsis* (Li et al. 2020), however, the specific function of nocturnal stomatal conductance (*g*_s_N) is not clear, but an adaptive role has been proposed by several authors which basically centres on plant functional response to environmental conditions, this is, partially open stomata during the night-time allow the plant to adapt to climate or soil nutrient conditions and improve early morning photosynthesis (Caird et al. 2007; Scholz et al. 2007; Easlon et al. 2009) while daytime stomatal conductance (*g*_s_) regulates photosynthesis (Farquhar and Sharkey 1982; Roche 2015). Nocturnal stomatal conductance dynamics show maximum levels before dawn (Hetherington and Woodward 2003; Caird et al. 2007) with a cyclic behavior which has been linked to the circadian plant response (Resco de Dios et al. 2013; Chowdhury et al. 2022). The biological clock-regulated response is thought to be an important plant adaptive trait, optimizing further fast and fine-tuned plant physiological adjustments to daily environmental cycles (Dodd et al. 2005; Vaze and Sharma 2013; Resco de Dios et al. 2016).

When investigating plant water losses, the stomatal conductance peak just before sunrise (pre-dawn *g*_s_) is especially relevant. This circadian pre-dawn response has been suggested as an advantageous adaptive trait for drought tolerance in wheat (Schoppach et al. 2020) and it has been shown that this capacity in Eucalyptus genotypes confers further gains in carbon assimilation (Resco de Dios et al. 2016). In general, higher chlorophyll content and aerial biomass have been associated with the plant’s ability to match intrinsic cyclic processes to cyclic environmental conditions (Pittendrigh et al. 1960; Dodd et al. 2005). Carbohydrate accumulation, especially starch, has been proposed as a mechanism underlying the rise in pre-dawn conductance (Easlon and Richards 2009), while other studies highlight the relevance of the circadian regulation of root hydraulic conductivity as a main driver of increased pre-dawn conductance (Caldeira et al. 2014). A better understanding of plant’s response to night-time temperatures in terms of their stomatal conductance may lead to the further identification and development of heat-resilient wheat. Achieving this goal necessitates the introduction of traits specifically tailored to the targeted environmental conditions. Therefore, the objective of this study was to characterize stomata morphology in a set of spring wheat grown under high night-time temperatures and high-yielding conditions, and investigate whether an increase in early daytime stomatal conductance provides an advantage to cope with heat stress. The hypotheses of this experiment were: (i) genetic variability for stomata morphometric attributes exists across a set of 12 elite wheat genotypes, and (ii) elevated morning conductance compensates yield (Yld) penalties derived from increased night-time temperature. Little is known about how diversity in morning conductance response translates into Yld gains or if this attribute is a residual response of the plant to night-time conditions.

## Materials and methods

### Germplasm and growth condition

**Figure 1.**
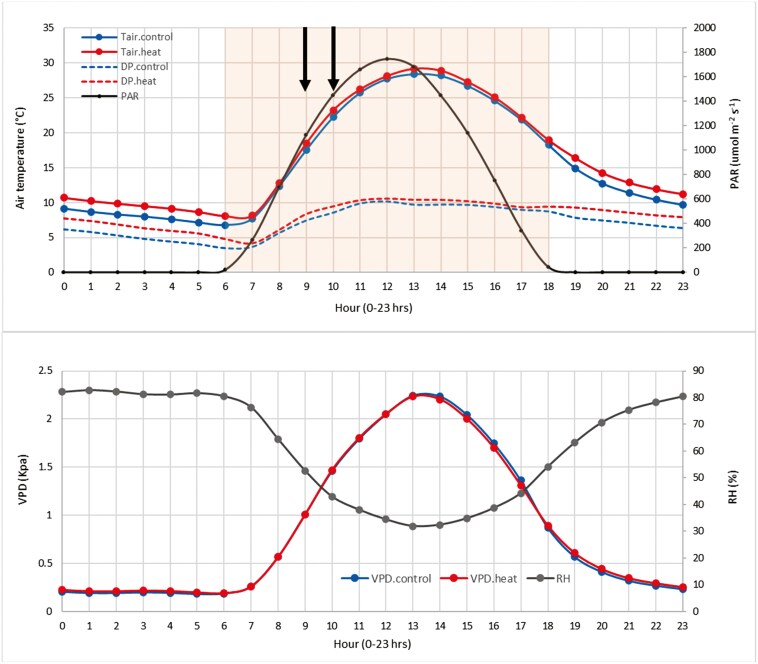
Meteorological conditions shown are the hourly averages for the period between the beginning of the heat treatment (19 February 2021) and the end of *g*_s_ measurements (31 March 2021). A) Tair: air temperature for the control (Tair.control) and heat treatment (Tair.heat). DP: is the dew point temperature for the control (DP.control) and heat treatment (DP.heat); PAR: photosynthetic active radiation; Black arrows indicate the time of *g*_s_ measurements. B) VPD: canopy temperature-based vapour pressure deficit for the control (VPD.control) and heat treatment (VPD.heat). RH: relative humidity.

The experiment consisted of single flat beds arranged in a complete randomized block design with two replicates for each of the two treatments: nocturnal high temperature and control exposed to ambient conditions. Nocturnal heat treatment consisted of the exposure of wheat canopies 2°C above the control canopies between 18:00 and 6:00 h starting from the beginning of the booting stage and ending at the beginning of physiological maturity. This period of time was chosen based on photosynthetic active radiation (PAR) records (≈ 0) for the experimental site, as shown in Fig. 1. The wheat genotypes were sown in the field in 0.5 × 3.2 m plots in December 2020 and harvested in April 2021; irrigation was applied with a drip system every 15 days on average to avoid water limitations, with a total of 400 mm distributed across the whole season. Fertilization, herbicides, and fungicides were applied as required. Each treatment included two field replicates containing 12 elite wheat genotypes which reported contrasting heat tolerance in previous field studies performed at CIMMYT on late sowings (Table 1). During the crop season, the average minimum/maximum temperatures were 8.2/29.0°C and 9.1/29.5°C for the control and heat treatment, respectively. During the nocturnal heat treatment period, the difference between the average canopy temperatures recorded in the heat and the control plots was ≥ 1.8°C on 82% of the nights (McAusland et al. 2023). Meteorological data collected on the experimental site is presented in Fig. 1.

**Table 1.** List of 12 elite wheat genotypes evaluated under high night-time temperatures in the Yaqui Valley, Sonora, during the season 2020–2021. The heat tolerance column classification was made based on previous studies performed under heat stress on delayed sowings at CIMMYT which resulted in elevated day and night-time air temperatures during the crop season.

Genotype number	CIMMYT GID	Cross name	Heat tolerance
1	5077000	CIRNO C 2008	Intermediate
2	2465	PAVON F 76	Tolerant
3	7806808	BORLAUG100 F2014	Intermediate
4	5893282	WEEBILL1	Low
5	3825355	SOKOLL	Tolerant
6	3855011	VOROBEY	Tolerant
7	3823821	PASTOR//HXL7573/2*BAU	Low
8	7129721	SOKOLL//PUB94.15.1.12/WBLL1	Tolerant
9	5865670	BAV92/SERI	Tolerant
10	5180627	BAV92/SERI	Low
11	6692380	PUB94.15.1.12/FRTL/5/CROC_1/AE.SQUARROSA (205)//BORL95/3/PRL/SARA//TSI/VEE#5/4/FRET2	Tolerant
12	7171118	SAUAL/WHEAR//SAUAL/3/PBW343*2/KUKUNA*2//FRTL/PIFED	Low

### Nocturnal heating system

**Figure 2.**
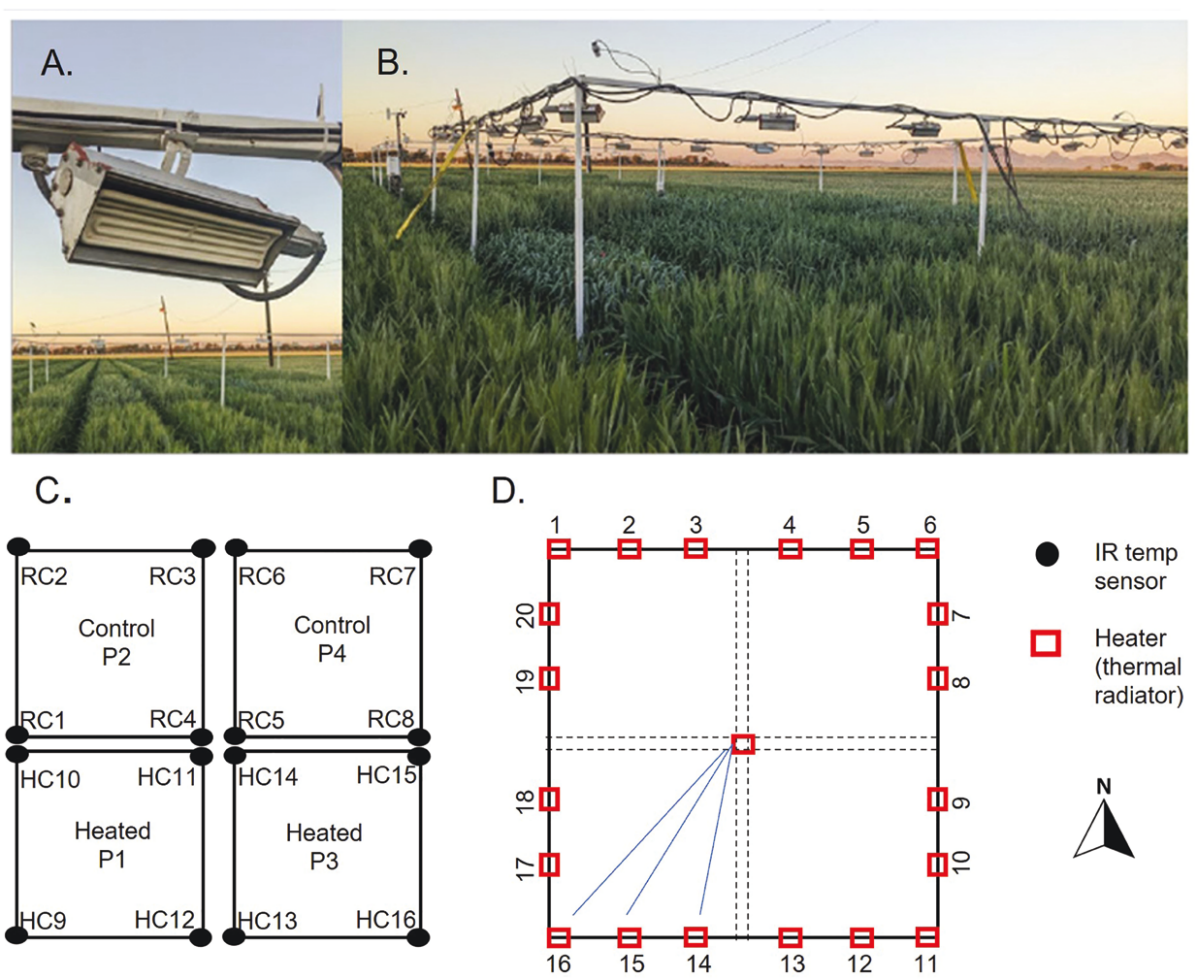
Diagram of the experimental design and heating system. A) One of the 21 heaters located on each of the two heat blocks; B) metal structure of 7 × 7 m surrounding each of the four blocks on which the heating systems were set; C) The experimental design indicating with HC# and RC# the location of the infrared thermometers used to record the canopy temperature and adjust the heat to + 2°C. D) The location of each of the 21 heaters on the two heat blocks is indicated with squares (McAusland et al. 2023).

Nocturnal heating was applied utilizing 21 heaters (thermal radiators) per each of the two heat blocks (FTE-1000 model, 1,000W, 240 V, 245 mm long 60 widths, by Mor Electric Company Heating Association Inc. Comstock Park, MI, USA) which were distributed around the perimeter on two square metal structures of 7.1 × 7.1 m each, surrounding the blocks. During the crop season, the metal structure was raised according to plant height increments to ensure that the distance between the wheat plants and the heaters was 1.2 m (Kimball et al. 2015). The increment in nocturnal canopy temperature was controlled using four infrared thermometers (IRTS Apogee Instruments Inc., Logan, UT, USA) located on the four corners of each of the four blocks (two heated, two control) at 45° of inclination relaying on the proportional, integrative, and derivative routine described in Kimball et al. (2015) which was used to maintain the heated block 2°C above the temperature recorded in the control blocks. Canopy temperatures were automatically collected every 15 min by a datalogger (CR1000 Campbell Sci, Inc., Logan, UT, USA) (Fig. 2).

### Stomatal conductance

Stomatal conductance was measured in the morning after dew had completely evaporated from wheat canopies at 9:00 am and repeated at 10:00 am (*g*_s_D9 and *g*_s_D10, respectively) on the adaxial leaf side, and during the night period between 19:00 and 21:00 h (*g*_s_N) before dewfall on the adaxial and abaxial leaf sides, both during the booting and grainfilling stages (heading + 12 days) on four flag leaves per experimental plot. Due to technical limitations associated with dew deposition, the stomatal conductance was not recorded during the pre-dawn period o after 21:00 h. Porometers Li-600 (Li-Cor, Lincoln, NE, USA) were used with the standard settings for daytime measurements suggested in the instrument manual, except for the resolution which was set to 0.005 mol m^-2^ s^-1^. Mean stomatal conductance by stage was calculated across times and genotypes and is presented as *g*_s_.B and *g*_s_.G, for the booting and grainfiling stages, respectively. The mean stomatal conductance across the stage and time of the morning is presented as *g*_s_.Av.

### Stomatal morphometric attributes

**Figure 3.**
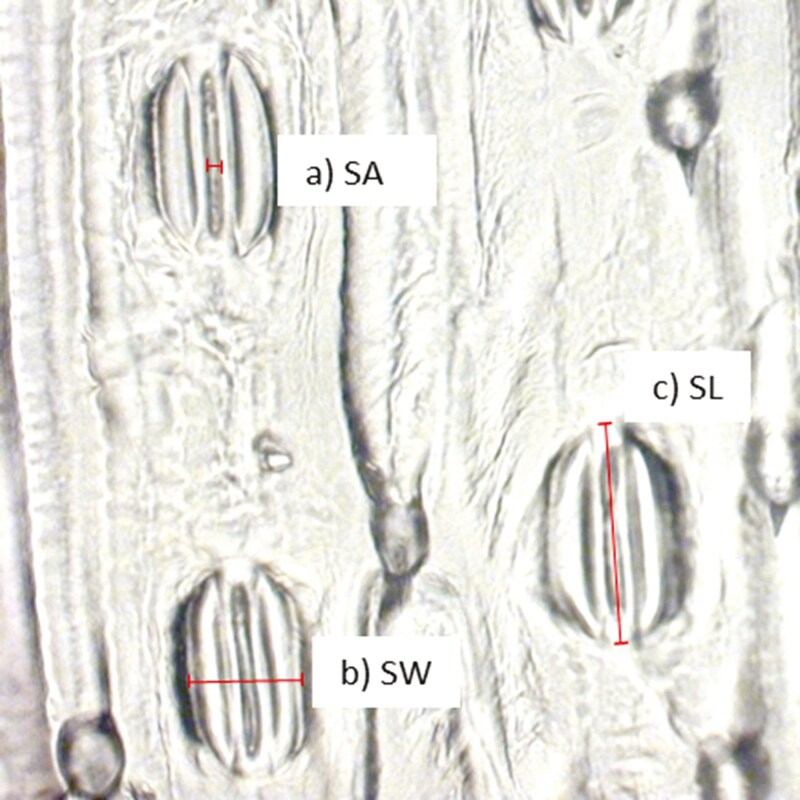
Stomata morphological characteristics a) SA: stomatal aperture, b) SW: stomatal width, c) SL: stomatal length.


Diurnal and nocturnal stomatal prints were taken for the set of 12 wheat genotypes during the grain-filling stage (heading + 12 days) on the adaxial and abaxial leaf sides. Stomatal prints were collected on attached leaves using transparent nail polish (Hilu and Randall 1984; Wu and Zhao 2017) between 9:00 and 10:00 h and between 19:30 and 20:30 h. In the field, a generous layer of polish was applied to the middle section of two attached flag leaves per plot, avoiding veins on the adaxial and abaxial leaf side, and let dry for at least 1 h. After complete drying, the nail polish with the stomata prints was carefully removed with tweezers and located on a microscope glass slide. A second microscope glass slide was located over the nail polish print and secured with transparent tape on the borders. The stomatal length (SL), stomatal width (SW), and stomatal aperture (SA) were measured on Image J, using images collected from a microscope Zeiss Axio imager.M2 with an Axio Vision AxioCAM MRc at 40 × (Fig. 3). Stomatal density (SD) was calculated as the number of stomata by mm^2^. A total of 192 stomatal prints were collected; stomatal counting to obtain SD was performed in different fields of view by quadruplicated on each of them making a total of 731 counts; for each of the later, three random stomata were measured to record SL, SW, and SA which resulted in a total of 2127 stomata measured (excluding missing data or damaged samples). Stomatal size was calculated by multiplying the measurements of SL by SW on nocturnal imprints as an approximation of guard cells length and width; this, to ensure that stomata were as closed as possible (Franks and Beerling 2009). The stomata ratio (SR) was calculated as the SD of a given leaf side divided by the total SD of both sides (Muir 2018). Maximum diffusive conductance to water vapour (*g*_s_max) was estimated according to Equation 1:

$$\begin{array}{l} g_{s}max=\frac{d}{v}.\;SD.a_{max}\;/\;(\;p+\;\frac{\pi }{2}\;\sqrt{\frac{a_{max}}{\pi }} \end{array}$$ (1)

where *d* is the diffusivity of water vapour in air (m^2^ s^-1^), *v* is the molar volume of air (m^3^ mol^-1^) at 20°C, the average temperature recorded during the period of prints collection; *p* is the pore depth (µm) assumed to be equivalent to guard cell width at the middle of the stomata (Franks and Beerling 2009; Lundgren et al. 2019) and *a*_max_ is the maximum area calculated assuming an eliptic shape as π × SL/2 × SW/2. All these traits were calculated for the adaxial and abaxial leaf side.

### Yield and yield components

Grain yield (Yld), biomass (Bm), grain number (Gn), and thousand-grain weight (TGW) were calculated at harvest according to Pietragalla et al. (2012) and Pask et al. (2012). Plant phenology was recorded as the number of days after emergence (dae) required to reach anthesis (DTA) and physiological maturity (DTM), corresponding to the day when 50% of the plot reached Zadoks decimal growth stage 6.0 and 9.0, respectively (Zadoks et al. 1974).

### Statistical analysis

Statistical analyses were performed in R (R Core Team 2023). Normal distributed traits such as the morphological stomatal measurements were analysed using a mixed linear model, while the not-normal distributed traits such as the stomatal conductance were analysed using a robust analysis of variance test (package WRS2) with an *α =* 0.05 (Wilcox 2003). Two groups of outstanding genotypes were contrasted for their agronomic performance utilizing a *t*-test. A principal component analysis (PCA) was performed to identify the main components that capture most of the variability under heat stress and the control.

## Results

### Stomatal conductance

**Figure 4.**
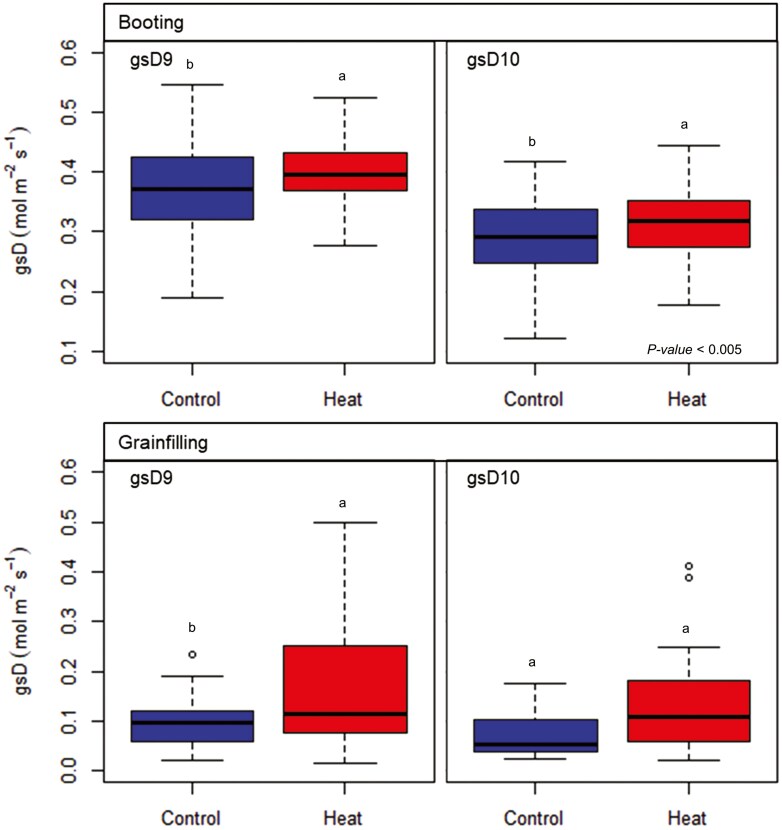
Early daytime stomatal conductance measured at 9:00 (*g*_s_D9) and 10:00 h (*g*_s_D10) during the booting and grain-filling stages in 12 elite wheat genotypes grown under high night-time temperatures. Heat indicate data for the high night-time temperatures treatment (+ 2°C). The lower and upper borders of the boxplots correspond to the first and third quartiles, the black lines within the boxes indicate the median, the dotted lines correspond to the minimum and maximum, while outliers are indicated with an empty dot (For each boxplot *n* > 22, 2 reps for a total of 12 genotypes).

Early daytime stomatal conductance, *g*_s_D9 and *g*_s_D10, measured during booting were significantly higher in the heated than the control plots (*P* < .005); mean *g*_s_D9 and *g*_s_D10 for heated plots were 7.2 and 8.1% higher than in control plots, respectively (Fig. 4). During the grain filling, *g*_s_D9 was significantly higher (71%) in the heated plots (*P* < .05); and *g*_s_D10 was still higher (63%) in the heated plots (*P* = .054). Over both growth phases, stomatal conductance decreased with the time of the day showing that *g*_s_D10 (0.278 ± 0.10 mol m^-2^ s^-1^) was lower than *g*_s_D9 (0.307 ± 0.16 mol m^-2^ s^-1^) when combined across treatments (*P* < .05).

Genotypic variability was observed for *g*_s_D9 and *g*_s_D10 during the booting stage (*P* < .05); genotype GID-3823821 showed the highest early daytime stomatal conductance under both, heat and control conditions. However, larger differences between genotypes were found in the control; genotype GID-3823821 showed up to 46% higher stomatal conductance than other genotypes; the minimum *g*_s_D9 and gsD10 in the control plots were 0.301 ± 0.10 and 0.242 ± 0.07 mol m^-2^ s^-1^ reported by genotypes GID-5865670 and GID- 3825355, respectively, while the maximums were 0.440 ± 0.07 and 0.346 ± 0.05 mol m^-2^ s^-1^ both reported by GID-3823821.

Irrespective of leaf side, phenological stage or genotype, nocturnal stomatal conductance was significantly lower under heat conditions (*P* < .001). Average *g*_s_N across all genotypes was 52.3% lower in the heat treatment (*P* < .001), with means of 0.035 ± 0.47 mol m^-2^ s^-1^ and 0.017 ± 0.020 mol m^-2^ s^-1^, for the control and heat treatment respectively. Reductions in *g*_s_N by the effect of the increased night-time temperatures were larger on the abaxial leaf side showing 60.1% less *g*_s_N, while on the adaxial *g*_s_N was reduced only by 43.6% when compared to the control. However, the extent of *g*_s_N reduction due to the exposure to high night-time temperatures is highly dependent on the genotype (*P* < .001); genotype GID-5893282 showed that *g*_s_N from the adaxial and abaxial leaf side were reduced by 75 and 84% when compared to the control, while GID-7171118 only reduced *g*_s_N by 5.7 and 1% on the adaxial and abaxial leaf side, respectively. Independently of the treatment or leaf side, *g*_s_N increased with leaf age (*P* < .0001); during the booting and grain-filling stages *g*_s_N mean was 0.020 ± 0.028 and 0.040 ± 0.048 mol m^-2^ s^-1^, respectively, and the adaxial leaf side showed 28% higher *g*_s_N than the abaxial leaf side (*P* > .05). Additional details of *g*_s_N can be found on McAusland et al. (2023).

### Stomatal morphometric variability

**Figure 5.**
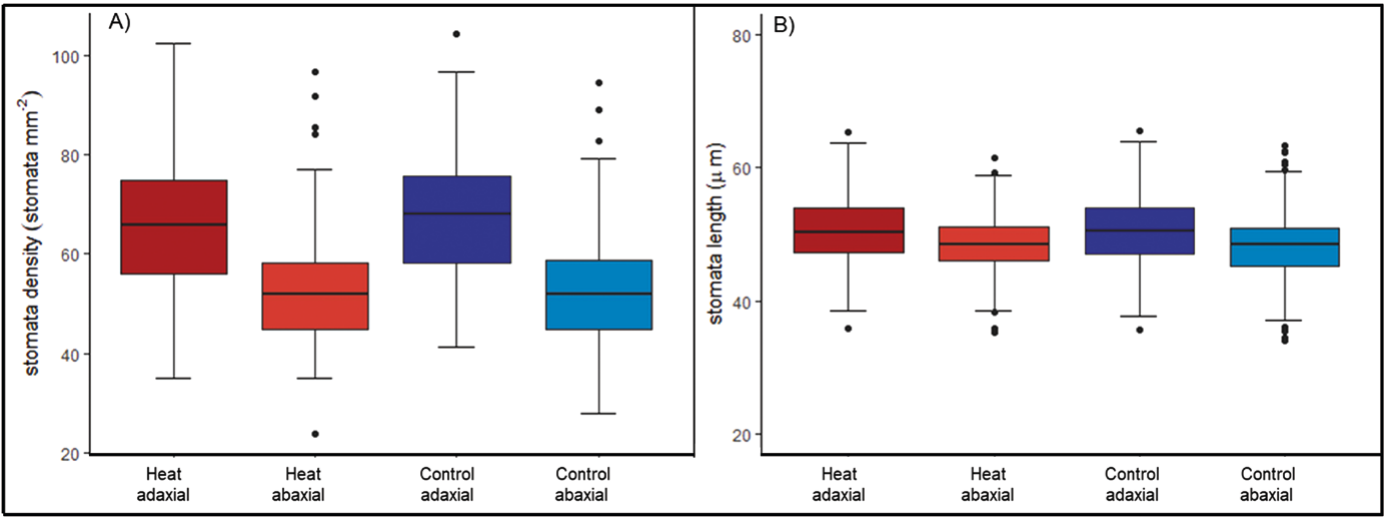
Comparison of the abaxial and adaxial leaf sides SD and SL recorded in 12 elite wheat genotypes across treatments.

**Figure 6.**
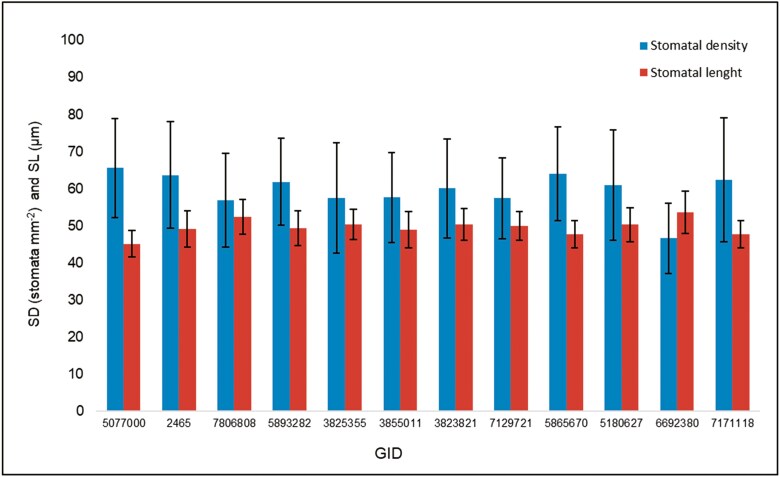
Stomatal density (SD), and stomatal length (SL), recorded on the adaxial leaf side of 12 elite wheat genotypes grown under high night-time temperatures. Standard deviation bars are indicated on the top of each column. (Details on genotypes are available in Table 1).

**Figure 7.**
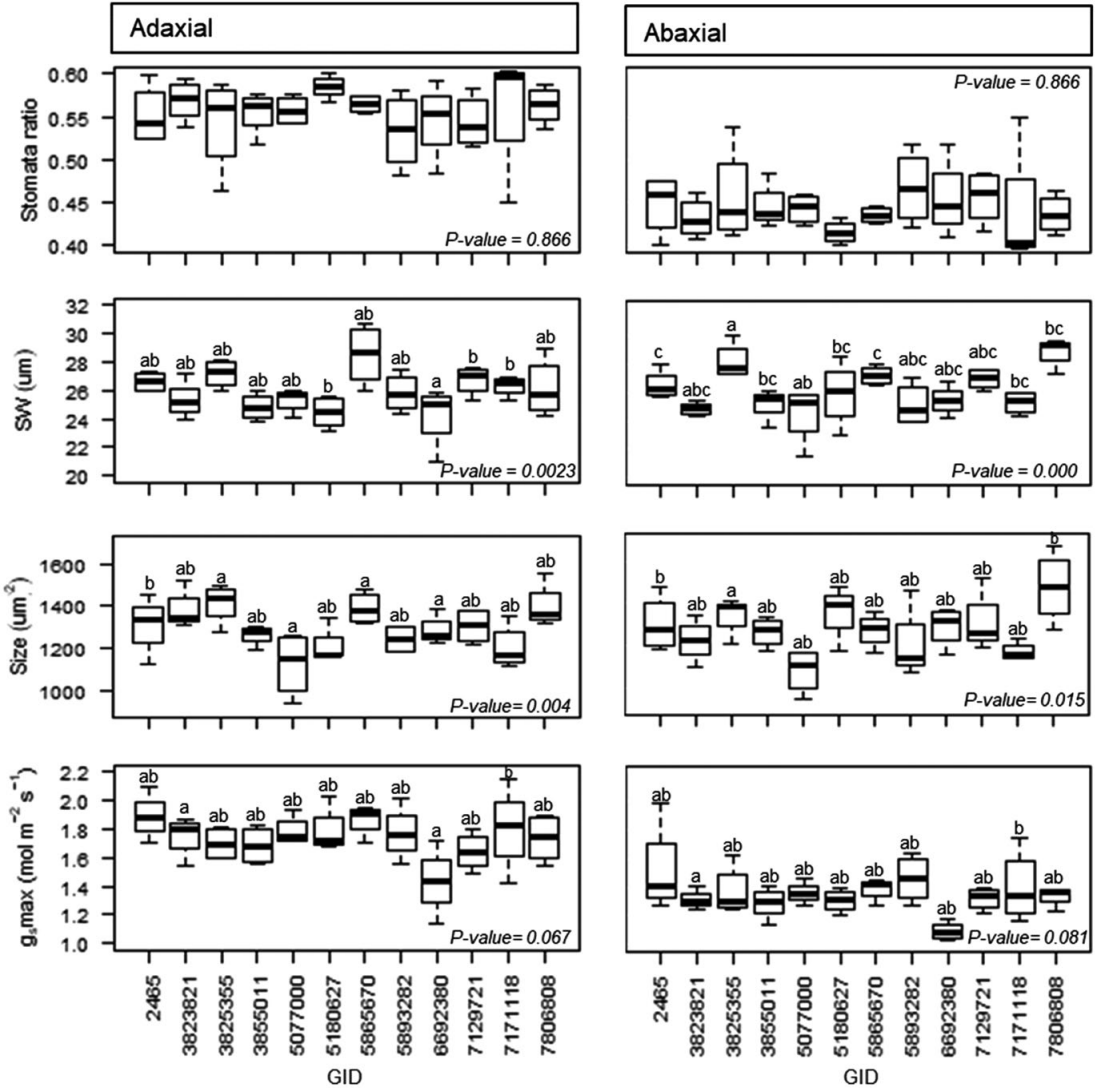
Comparison of the stomata morphological attributes recorded on the adaxial and abaxial leaf side of the flag leaf of 12 elite wheat genotypes, and the maximum stomatal conductance estimation (*g*_s_max). Letters above the boxes indicate the means comparison results between genotypes at the specific *P*-value obtained from the GLMM analyses. For stomata ratio (SR) and stomata width (SW), the data shown are means across day/night records and phenological stages with two biological replicates; for Size only nocturnal records were averaged across phenological stages, and for *g*_s_max only daytime records were averaged across phenological stages.

Comparing leaf sides, stomata were found to be larger but not wider on the adaxial leaf side (*P* < .001). Whereas on the adaxial leaf side, the mean SL was 50.6 ± 5.3 µm, the mean length on the abaxial leaf side was 48.4 ± 4.2 µm. SD was also higher on the adaxial leaf side (*P* < .001); observations showed 67 ± 13 stomata mm^-2^ on the adaxial leaf side while on the abaxial leaf side, the mean was 53 ± 10 stomata mm^-2^ (Fig. 5). Genotypic differences were observed for SD and SL (*P* < .001); genotypes GID-6692380 and GID-7806808 showed the largest stomata on both leaf sides and the lowest stomata densities; opposite, genotype GID-5077000 showed the lowest SL and the highest SD together with genotype GID-5865670 (Fig. 6). The SW on the adaxial leaf side were observed on GID-5865670 and the tightest on GID-3855011, GID-5180627, and GID-6692380 (*P* = .02, Fig. 7); on the other hand, GID-7806808 showed the widest stomata while GID-3823821 and GID-5077000 showed the tightest on the abaxial leaf side (*P* = .002, Fig. 7).

Significant differences between genotypes were observed for mean stomata size from the adaxial and abaxial leaf sides (*P* < .05). GID-7806808 showed the biggest and GID-5077000 and GID-7171118 the smallest (*P* = .015, Fig. 7). These results were replicated for the bottom where the same GID highlighted as the biggest and smallest (*P* = .004). The mean SR for the adaxial and abaxial leaf side was 0.56 + 0.036 and 0.46 ± 0.36, respectively, but no genotypic variability was observed for the trait (Fig. 7). Anatomical maximum stomatal conductance to water (*g*_s_max) between genotypes resulted equal independently of the leaf side, however, the adaxial reported 28% highest *g*_s_max than the abaxial (*P* < 2.2e-16).

SA varied with temperature growing conditions. In the morning, plants under heat showed larger SA than those from the control independently of the leaf side (*P* < .0001). However, when comparing the adaxial and abaxial SA, the difference between treatments was more evident on the adaxial leaf side. The mean SA on the adaxial leaf side under heat was 4.4 ± 1.9 µm while under control the mean SA was 3.4 ± 1.3 µm; on the other hand, the abaxial leaf side showed a mean SA of 3.7 ± 1.4 µm and 3.4 ± 1.1 µm for the heat and control plots, respectively. Differences between leaf sides were significant (*P* < .05); the adaxial leaf side stomata were 10% more open in the abaxial leaf side reaching up to 18% under heated conditions. SA during the night-time was smaller in the heat treatment (2.08 ± 0.84 µm) compared to the control (1.91 ± 0.67 µm), irrespective of the leaf side (*P* < .001). Even though night-time SA on the abaxial leaf side was slightly larger, no statistical differences were observed in comparison to the SA of the adaxial leaf side (*P* > .05).

### Agronomical performance

The mean Yld for heat and control were 465 ± 59 gm^-2^ and 443 ± 57 gm^-2^, respectively; no statistical differences between treatments were detected for this trait. However, genotypic variability was significant under heat (*P* < .001); the maximum Yld was 588 gm^-2^ reported by genotype GID-5077000, while the lowest Yld was 380 gm^-2^ reported by genotype GID-6692380. In agreement, genotypes GID-5077000 and GID-6692380 also showed the highest and lowest Gn and Bm, respectively. Maximum TGW under heat was 47.7 g reported by genotype GID-5865670. Genotypic variability for Yld was observed also under control conditions (*P* < .001); the maximum and minimum Yld under control were reported by genotype GID-3823821 (557 gm^-2^), GID-2465 and GID-6692380 (365 and 388 gm^-2^, respectively). Bm under control conditions fluctuated between 980 and 1332 gm^-2^, reported by genotypes GID-5077000 and GID-5180627, respectively; while the maximum Gn (14 318 grains) was reported by genotype GID-5893282.

Anthesis was recorded at 81 ± 2.7 and 82 ± 2.9 dae for heat and control, respectively. Maturity was recorded at 115 ± 1.5 and 116 ± 1.1 dae for heat and control, respectively. No statistical differences were found between treatments for DTA and DTM.

### Relationship between the stomatal attributes, stomatal conductance, and agronomic parameters

**Figure 8.**
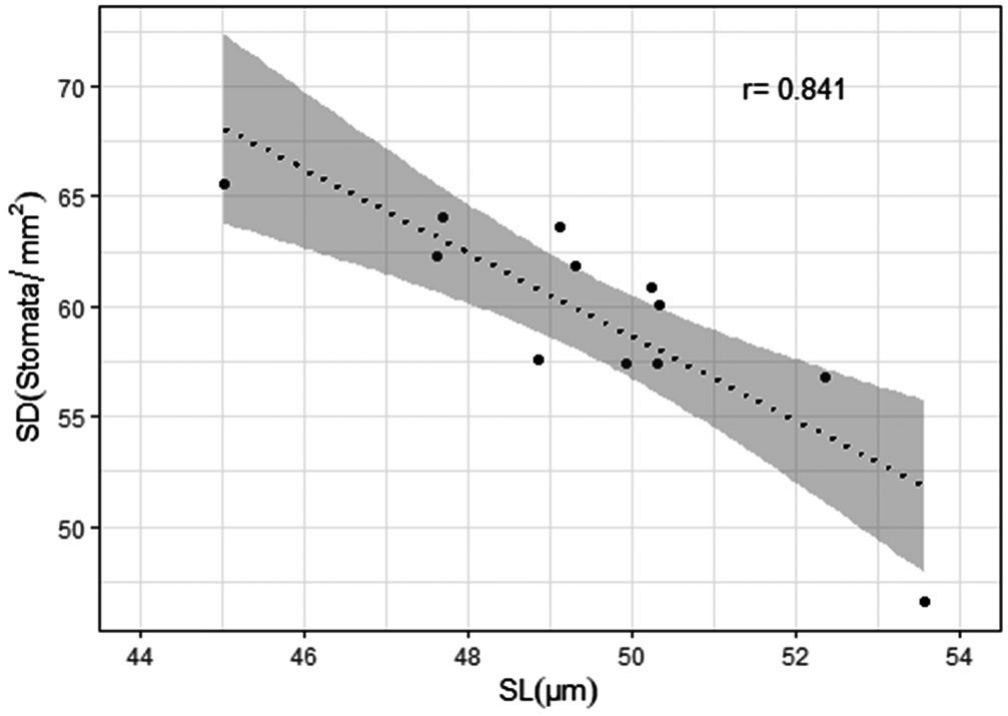
Negative correlation between stomatal length (SL) and stomatal density (SD) for a set of elite wheat genotypes grown under high night-time temperatures. The means presented are the combined average for the adaxial measurements recorded on 12 elite wheat genotypes across the control and heat treatment. In grey shadow the confidence interval at α = 0.05.

**Figure 9.**
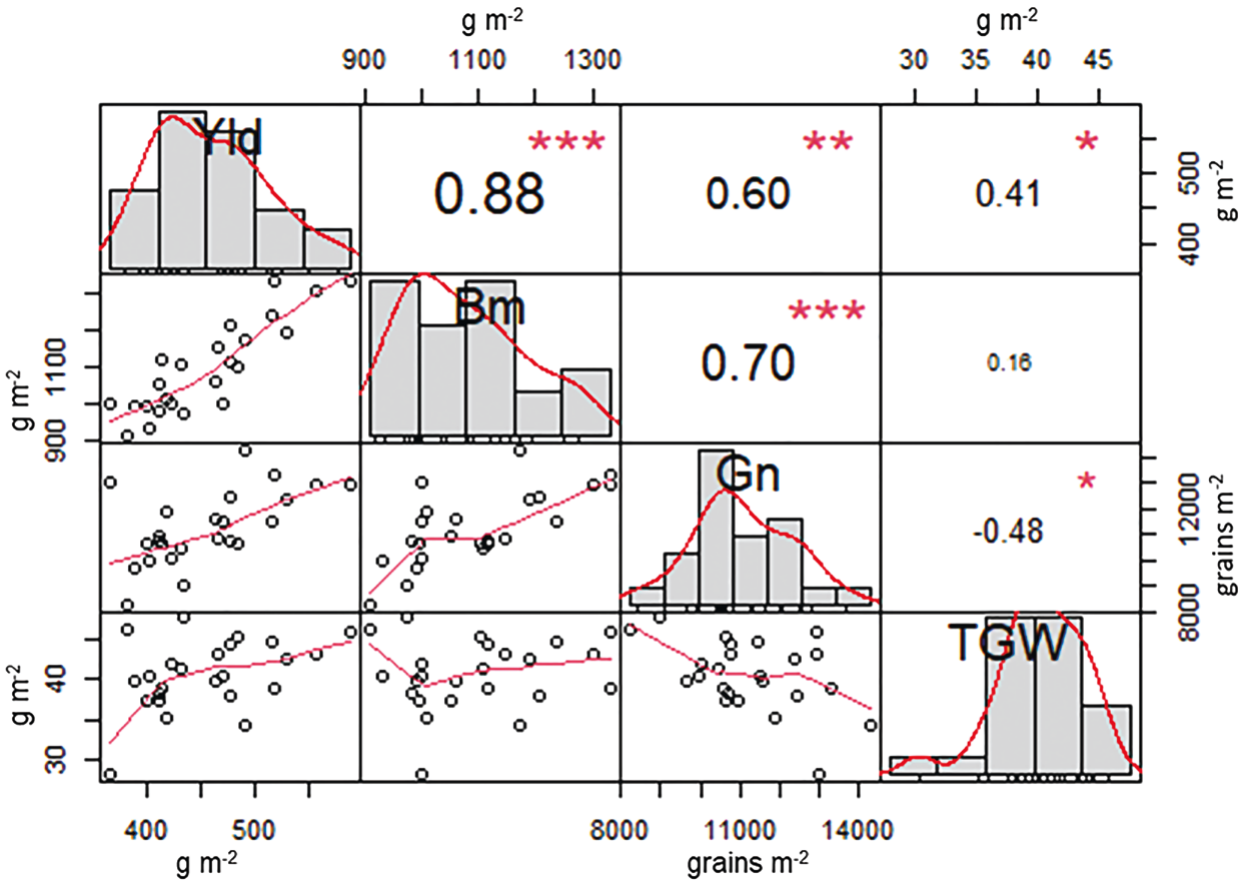
Pearson correlation coefficient between grain yield (Yld), Biomass (Bm), Grain number (Gn), and thousand-grain weight (TGW) for 12 elite wheat genotypes grown under control and high night-time temperature treatment. **: *P* < .01; ***: *P* < .001.

The combined analysis across treatments and genotypes showed that SD and SL were strongly and negatively associated (Fig. 8, *r* = 0.84, *P* < .001). Higher stomatal conductance observed under the heat treatment, mirrored larger SA (*P* < .0001), however, no significant correlations were found between SD, SL, SW, or SA and stomatal conductance (*g*_s_D9 or *g*_s_D10) when the Pearson correlation coefficient was calculated. The average stomatal conductance across stages and time (*g*_s_.Av) was found to correlate with Yld (0.45, *P* < .05). High and positive correlations were observed between Yld, Bm, and Gn (Fig. 9, *P* < .01). Gn was negatively correlated with SD (*r *= −0.56, *P* < .001).

**Figure 10.**
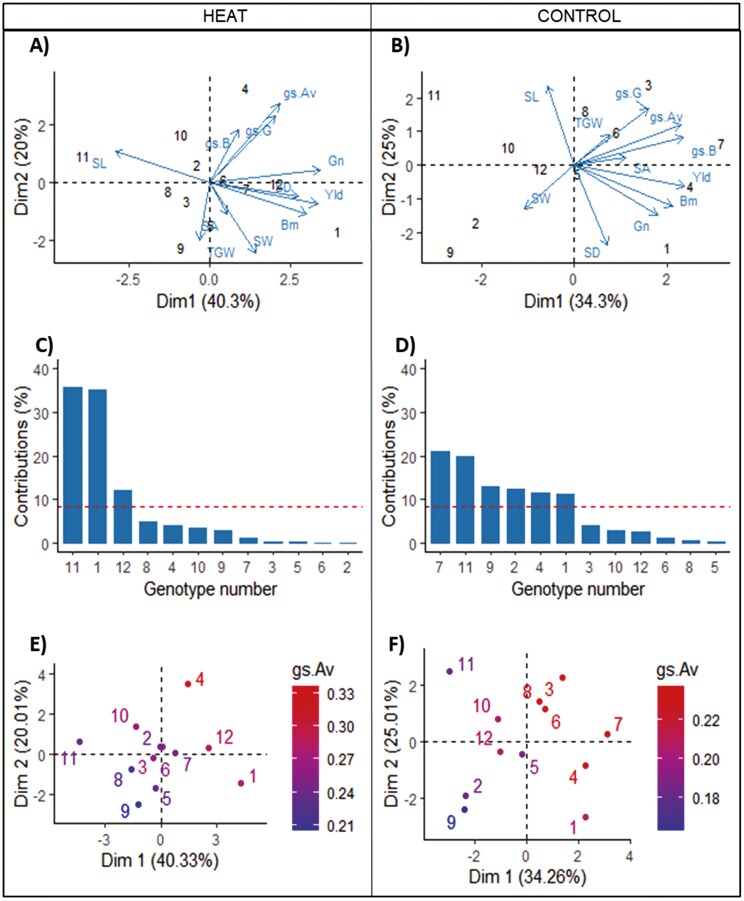
Principal components analyses for stomatal conductance, Yld, and Yld components recorded in a set of 12 elite wheat genotypes grown under high night-time temperature (A), and control conditions (B). Contribution of the best-represented genotypes to PC1 under heat (C) and control (D) conditions, where the dashed line represents the expected value if the contributions were uniform for all the genotypes. Average stomatal conductance (*g*_s_.Av) values for the 12 elite wheat genotypes grown under high night-time temperature (E), and control conditions (F). *g*_s_.B: average stomatal conductance during the booting stage; *g*_s_.G: average stomatal conductance during the grain-filling stage SL: stomatal length; SD: stomatal density; SW: stomatal width; SA: stomatal aperture; Yld: yield; Bm: biomass; Gn: grain number; TGW: thousand-grain weight. The genotype number is presented instead of the CIMMYT GID for space reasons.

Two PCA analyses performed by treatment grouped the genotypes and traits collected under heat and control, respectively, into two major components explaining over 60.3 and 59.3% of variation each (Fig. 10). Under heat, the genotypes better represented and explaining the highest variance of principal component one (PC1) were genotypes GID-6692380 and GID-5077000 (35.9 and 35.3%, respectively); genotypes better represented and explaining the highest variance of PC2 were genotypes GID-5893282 and GID-5865670 (46.2 and 23.4%, respectively). SL had the highest positive loading to PC1, while Yld, Gn, and Bm had the highest negative loading. SW had the highest positive loading to PC2, while *g*_s_.B had the highest negative loading. PC1 had an eigenvalue of 4.4 and accounted for 40.3% of variation; PC2, had an eigenvalue of 2.2 and accounted for 20% of variation. The angle between vectors indicated high and positive correlations between Yld, Bm, and Gn under heat. Under control conditions (Fig. 10), the genotypes better represented and explaining the highest variance of PC1 were GID-3823821, GID-6692380, and GID-5865670 (21.1, 19.9, and 13.1%, respectively); the genotypes better represented and explaining the highest variance of PC2 were GID-5077000 and GID-6692380 (21.1 and 19%, respectively). SW and Yld had the highest positive and negative loadings to PC1, respectively. SD had the highest positive loading to PC2, while SL had the highest negative loading. PC1 had an eigenvalue of 3.8 and accounted for 34.3% of variation; PC2, had an eigenvalue of 2.8 and accounted for 25% of variation. In concordance with the heat treatment, the angle between vectors indicated high and positive correlations between Yld, Bm, and Gn under control conditions.

**Figure 11.**
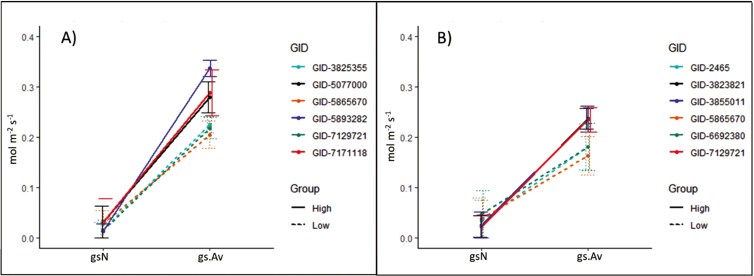
Change in stomatal conductance from night-time (*g*_s_N) to early morning (*g*_s_.Av) recorded for the top three (High) and bottom three genotypes (Low) with highest and lowest *g*_s_.Av. A) High night-time temperature treatment and B) Control conditions. Bars indicate the standard error. Only genotype GID: 5865670 appears on both treatments.

Genotypes that steadily showed high *g*_s_.Av also showed higher Bm, Gn, and Yld (Table 2). To determine the improvement derived from selecting germplasm based on their early stomatal conductance, the three highest and three lowest *g*_s_.Av genotypes by treatment were grouped into two categories ‘High’ or ‘Low’ (Table 2). *T*-test comparing groups, showed significant differences within groups for Yld, Gn, and *g*_s_.Av (*P* < .05). Under heat, the ‘High’ group reported 14% higher Yld, 8.8% higher Bm, and 13.7% higher Gn than the mean for the treatment of 12 genotypes. Under control conditions, gains were moderate, the ‘High’ genotypes showed around 4% higher Yld and Bm than the mean for the treatment of 12 genotypes. Early daytime conductance (*g*_s_.Av) was 35 and 43% higher in the ‘High’ compared to the ‘Low’ group in the control and heat treatment, respectively (12.5 and 8.2 times higher than *g*_s_N for the ‘High’ and ‘Low’ groups, respectively), leading Yld increments of 19% under both, heat and control. Only genotype GID-5865670 was consistently labelled as ‘Low’ under both treatments (Fig. 11). For this set of genotypes *g*_s_.Av was found positively and significantly correlated with Yld (*r* = 0.60, *P* = .039).

**Table 2.** Genotypes with the highest and lowest early daytime stomatal conductance (*g*_s_.Av) on each treatment, grouped as ‘High’ or ‘Low’, and their nocturnal stomatal conductance (*g*_s_N), grain yield (Yld), biomass (Bm), and grain number (Gn).

CIMMYT GID	Group	*g*_s_.Av	*g*_s_N	Yld	Bm	Gn
		mol m^-2^ s^-1^	Mol m^-2^ s^-1^	gm^-2^	gm^-2^	grains
Heat						
3825355	Low	0.225 ± 0.016	0.016 ± 0.016	476 ± 10.2	1114 ± 32.4	10 779 ± 1156
7129721	Low	0.218 ± 0.021	0.015 ± 0.021	422 ± 11.7	997 ± 4.8	10 031 ± 210
5865670	Low	0.205 ± 0.027	0.028 ± 0.027	432 ± 177	973 ± 385	9005 ± 3406
5077000	High	0.279 ± 0.031	0.032 ± 0.031	588 ± 7.0	1331 ± 235	12931 ± 1808
5893282	High	0.336 ± 0.016	0.013 ± 0.016	470 ± 107	1001 ± 220	11 541 ± 1889
7171118	High	0.288 ± 0.046	0.032 ± 0.046	528 ± 48.5	1189 ± 11.0	12 372 ± 297
	Mean by:					
	Treatment	0.253 ± 0.036	0.024 ± 0.026	465 ± 59.0	1078 ± 130	10 806 ± 1364
	*Low group*	0.216 ± 0.010	0.020 ± 0.005	443 ± 28.7	1028 ± 75.0	9938 ± 891
	*High group*	0.308 ± 0.031	0.023 ± 0.015	529 ± 59.0	1166 ± 165	12 236 ± 699
Control						
2465	Low	0.181 ± 0.045	0.034 ± 0.045	365 ± 51.1	1001 ± 115	12 991 ± 1266
5865670	Low	0.163 ± 0.038	0.038 ± 0.038	432 ± 5.50	1106 ± 74.3	10 471 ± 437
6692380	Low	0.181 ± 0.047	0.047 ± 0.047	388 ± 90.3	990 ± 186	9651 ± 1409
3855011	High	0.236 ± 0.026	0.026 ± 0.026	413 ± 28.1	1115 ± 143	10 598 ± 721
3823821	High	0.237 ± 0.021	0.023 ± 0.021	557 ± 37.4	1303 ± 81.5	12 928 ± 1122
7129721	High	0.235 ± 0.024	0.022 ± 0.024	411 ± 36.4	1050 ± 142	10 974 ± 641
	Mean by:					
	Treatment	0.209 ± 0.027	0.040 ± 0.048	443 ± 57.4	1109 ± 122	11 722 ± 1436
	*Low group*	0.175 ± 0.010	0.040 ± 0.004	395 ± 33.8	1032 ± 64.0	11 038 ± 1741
	*High group*	0.236 ± 0.001	0.024 ± 0.003	460 ± 83.5	1156 ± 131	11 500 ± 1251

## Discussion

### Enhanced circadian regulation of *g*_s_ as an adaptive trait

Several studies have shown that stomatal conductance fluctuates during the night time, reaching its maximum immediately before dawn (Caird et al. 2007; Resco de Dios et al. 2013). An anticipated response to light seems to be an adaptive circadian-regulated capacity. When grown under high temperatures, plants make a trade-off between cooling and water losses but when VPD increases, the plant aims to compensate, with increased closure of the stomata, however, when water is not a limitation the plant can increase the transpiration rate to cope with the high water demands required to cool the plant and maximize leaf gas exchange.

Increments in Bm, Gn, and Yld linked to higher early daytime stomatal conductance suggest that carbon assimilation is increased during the same period, however, this theory is frequently based on the assumption that photosynthesis increments are an overcompensation mechanism to cope with the high degradation of assimilates during the night through respiration, but night-time respiration under the heat treatment decreased during the booting and heading stages, instead of increasing as expected suggesting that respiration acclimatize to high temperatures (McAusland et al. 2023). A decreased consumption of carbohydrates during the night through respiration could have contributed to higher Ylds (Pinto et al. 2017; Impa et al. 2019; Xu et al. 2021).

The ‘anticipation hypothesis’ as cited by Resco de Dios et al. (2016) where the endogenous timer regulates the metabolic processes to prepare the plant for predictable events such as dawn, seems to explain the high-temperature impact on *g*_s_. Results showed that stomatal conductance was higher at 9:00 than 10:00 h, under both, control and heat treatment which suggests a circadian regulation of *g*_s_; nevertheless, this seems to be a heat-stress residual response which has been enhanced by exposure to high night-time temperature given that, despite the increase in VPD from 9:00 to 10:00 h, higher water losses were observed on the heat-treated plots in the early daytime where VPD was the same for both treatments. The latter could indicate that after the period of increased night-time temperature, where the SA and water losses were reduced, the heat-exposed genotypes maximized their stomata aperture and increased their stomatal conductance in contrast to the not-treated genotypes. The degree of early daytime SA was largely dependent on genotype and leaf side, but the trend was consistent demonstrating that the impact of nocturnal heat stress can be detected during the first hours of daytime possibly as trade-off of the nocturnal heating which compensated for Yld penalties.

Resco de Dios et al. (2016) reported the circadian regulation of *g*_s_, showing that genotypes with high pre-dawn *g*_s_ also exhibited faster stomatal responses to light at sunrise. In the current study *g*_s_ in the early morning was measured after dew evaporation due to technical limitations, but early daytime stomatal conductance has been associated with pre-dawn *g*_s_ (Drake *et al.* 2013; Resco de Dios et al. 2016). More experiments are needed to confirm the applicability of this hypothesis for wheat crops under heat stress.

When analysing SA, it was interesting that high night-time temperatures resulted in equal reductions in SA from both leaf sides during the night, but the nocturnal heat treatment prompted different responses in morning stomatal SA. Results revealed that the adaxial leaf side exhibited stomata 29.4% more open than their counterparts from the control; while the stomata on the abaxial leaf side in the heat treatment only increased their SA by 8.8% compared to the control. The latter suggests an enhanced circadian response by the genotypes exposed to the heat treatment, and notably, stomata from the adaxial leaf side demonstrated a heightened *g*_s_ response to the plant’s biological clock. It is widely accepted that function of both leaf sides is not necessarily coupled and these findings could indicate that stomata on the adaxial exhibit better control of the guard cell’s turgor. Studies on cryo-sectioned leaves of wheat have shown that reaching the maximum SAs requires a significant reduction of cell osmotic pressure between guards and subsidiary cells (Franks and Faquhar 2007).

It is important to note that the increase in *g*_s_ seems to be transient given that measurements at midday did not show the same pattern. As reported in a previous study involving the same wheat genotypes evaluated in this research (McAusland et al. 2023), night-time increment shows no effect on *g*_s_ when measured during the period of maximum irradiance which coincides with observations on forest species (Resco de Dios et al. 2016). However, results from the current study suggest that early daytime or pre-dawn circadian regulation of stomata conductance is worthwhile to be considered as a valuable adaptive attribute for crop breeding.

### Stomatal morphological traits in response to high night-time temperature

The number and size of stomata, together with opening and closure movements, are the main regulating factors of plant water losses and CO_2_ income for photosynthesis. Our observations of stomatal morphology from leaf impressions showed stomata on both leaf sides of the flag leaves with remarkable differences in stomatal densities between the adaxial and the abaxial leaf sides. Studies on diploid, tetraploid, and hexaploid wheat also showed higher SD and higher genetic variability on the adaxial leaf side compared to the abaxial (Khazaei et al. 2010; Cabello-Cornejo et al. 2019). *Amphistomatous* leaves with stomata on both leaf sides are common on grass species and occur mainly on herbaceous plants such as wheat (Metcalfe and Chalk 1950). This attribute seems to be advantageous for enhancing leaf-atmosphere gas exchange, especially on thin leaves where the pathway to transport CO_2_ between the atmosphere and the chloroplasts is reduced, the temperature gradients inside the leaf are diminished limiting water condensation that could act as a barrier restricting CO_2_ diffusion (Drake 2019).

From the evaluated germplasm, some genotypes were identified for containing many small stomata rather than a few long stomata, for instance, genotypes GID-5077000 and GID-5865670 exhibited the highest SD and the lowest SLs, on both leaf sides; on the other hand, genotypes GID-6692380 and GID-7806808 showed a small number stomata by mm^2^ but of larger size. This was in agreement with results from stomatal size estimations where for example, GID-5077000 and GID-7806808 showed one of the smallest and biggest stomatal sizes (µm^-2^), respectively. Increasing the number of stomata per unit of leaf area is the basic strategy to increase the stomatal conductance, however, the stomatal size reduction together with an increase in SD can be more advantageous given that the reduction in stomatal size seems to come with the reduction in stomatal pore depth, which together with SD is a main driving trait of maximum stomatal conductance (Lawson and Matthews 2020). Furthermore, smaller stomata exhibit faster responses to environmental changes (Franks and Beerling 2009). This explains the observed negative correlation between SD and SL (Fig. 8) but surprisingly, no differences in the calculated anatomical maximum stomatal conductance (*g*_s_max) between genotypes were found in this study.

The distribution of stomata across the leaf lamina has been cited as an important factor controlling the stomatal conductance (McKown and Bergmann 2018); this distribution is rather heterogeneous as has been observed in diverse crops, and is believed to be linked to the natural variability of the network of vascular bundles limiting gaseous mobility (Mansfield et al. 1990). Our results showed reduced stomatal densities on the abaxial leaf side which corresponded with lower daytime and night-time stomatal conductance reported in the literature (McAusland et al. 2023).

A number of studies have shown increased SD associated with high stomatal conductance as observed here on genotypes GID-5893282, GID-3823821, and GID-5077000 (Cabello-Cornejo et al. 2019). A high number of stomata has also been related to increased chlorophyll a and b content under water-limited conditions, suggesting that more pigments are available for capturing light and increasing photosynthetic rates, which in the three genotypes listed above, resulted in high Bm and Yld (Table 2).

## Conclusions

Elevated night-time temperatures increased early morning stomatal conductance and reduced nocturnal conductance. Results from this study showed that significant gains in Bm, Gn, and Yld can be assessed by selecting genotypes with high early morning stomatal conductance. The latter confirms the value of early daytime conductance for increased wheat productivity, particularly in hot-irrigated environments and this seems to be linked to the circadian rhythm of plant response to cyclic events by which the plant anticipates environmental cues and alleviates Yld losses. Moreover, it was observed that better-performing genotypes generally exhibit higher stomatal densities but this attribute is genotypic-specific. More studies are required to confirm these findings and explore additional factors that influence the stomatal conductance of wheat crops, as well as the role of the cuticle conductance during the pre-dawn period. This study is one of the few attempts to unravel the relevance of trade-off *g*_s_ processes associated with nocturnal warming and its contribution to increasing wheat Yld potential.

## Supplementary Material

plae072_suppl_Supplementary_Materials

## Data Availability

The data is available at Zenodo: https://doi.org/10.5281/zenodo.14325447.
