# Supplementary material for: Wheat genotypes selected for their high early daytime stomatal conductance under elevated nocturnal temperatures maintain high yield and biomass
Source: AoB Plants. 2024 Dec 25;17(4):plae072. doi: 10.1093/aobpla/plae072 (PMC12230296; doi:10.1093/aobpla/plae072)
Supplement: plae072_suppl_Supplementary_Materials [file plae072_suppl_supplementary_materials.pdf]

Response to the editor/reviewers

The authors thank the valuable time the editor and reviewers dedicated to read and comment this manuscript. We are sure that your input will improve this work. Below are the responses (in blue) to the questions and comments provided.

Editor Comments

Mainly, (i) **details on statistical evaluation are missing or poorly presented** which rise the feeling that the results and conclusions are not warranted and reliable. Additional details on statistics are provided on the Materials and Methods section as well as on the figures and tables, as requested.

(ii) **The concept should be better framed by relevant testable hypothesis and clear replies**. In the present version, the manuscript is rather descriptive. We admit that this was recurrent plea from reviews, and therefore, we include a set of hypotheses that we hope guide and focus the aims of our work, the new text is located the end of the introduction.

(iii) **The timing of the nocturnal heat treatment** (from booting to ripening- maturity) **and the timing of stomatal conductance measurements** (very early night 19-20 h and 9 h, 10 h in the morning) seems arbitrary **and needs some solid reasoning or at least more frequent repetition** in one or several plants to show the diurnal cycle of gs. The reasoning behind these choices is now stated on the Materials and Methods section and supported with meteorological data collected in the experimental site (new Figure 1), which shows the nocturnal period and other relevant traits. As stated, the main constraint for choosing these times for the stomatal conductance measurements is due to the rapid and copious dew formation in wheat canopies from ~21 to ~ 9 h.

(iv) based on my experience, it is not reliable to measure stomatal aperture from leaf imprints and microscopic images. **Maximum stomatal conductance calculated from stomata dimensions would give more relevant information**. We thank the editor critique regarding the methodology to assess stomatal attributes from imprints and microscopic images, however we disagree, given that the methodology is currently widely used to assess stomatal length, width and aperture (Jashim et al., 2024; Crestani, et al., 2023; Wu and Zhao 2017, https://shorturl.at/sNC4e) and as shown in Figure 2, our images show clear fields of view to conduct measurements. However, we decided to follow the advice and calculate the maximum stomatal conductance which was added to the manuscript as $g_s$max, although no significant differences were found in our genotypes regarding this trait. (Figure 6)

(v) Strictly speaking, it is not stomatal conductance but **leaf conductance what you measured and which includes also the cuticle contribution to water loss**. It can contribute significantly especially to the night-time transpiration and it should be mentioned. Methods attempting to partition stomatal and cuticular conductance on the intact leaves were published recently although not on wheat (e.g. Marquez et al.: New Phytologist 2022, Santrucek: Annals of Botany 2022). Some of my specific comments are shown below. Perhaps, they could help you to improve the manuscript.

As stated in the text, gsN and gsD were indeed measurements of stomatal conductance as it is widely accepted to approximate with a leaf porometer (Li-600, Li-Cor, Lincoln,

NE, USA). We do agree with the Editor of AoB that this would have been a good addition to our work. Unfortunately, we do not have fresh material, to design a set up to calculate cuticular conductance for our experimental plants since fresh materials along with careful determination of key environmental variables to assess this trait.

Unfortunately, although this is an interesting and timely topic, I cannot recommend your manuscript for publication in Annals of Botany for the reasons mentioned above.

We still thank the time and effort on handling our manuscript and have decided to do a transfer to *AoBplant* as you kindly recommend.

P3L12: Mizokami et al, 2022 - citation is missing in references. Added
P5L9-10: the same for works of Scholz, Huang and Yu. Added
P6L21: '18:00 and 6:00 hours': Is this period considered as night? What was irradiance especially at the beginning and end of the night? Text edited to provide meteorological information and a new figure (Figure 1) was included that shows PAR for the period between 18:00-6:00 hours.
P8L4-5 'during the night between 19:00 and 21:00 hours' Is this time period representative for the whole night? Apparently, it is assumed that the stomatal conductance is constant during the night period. Stomatal conductance likely changes during the night-time; however, technical limitations did not allow us to carry measurements of gs beyond 21:00 hours when dew was detected on the leaves and erratic readings were noticed in the porometers. We were very careful on detecting wet leaves before or after the measuring periods to avoid bias at the time of porometer measurements.

P8L6: It is stated here, that only on the adaxial leaf side was measured. However, in Results also the abaxial stomatal conductance is mentioned. Text edited to be clear, that gs during the morning (gsD) was measured only on the adaxial leaf side, and gsN on both leaf sides.

P8L22: I am sceptical about the measurements of stomatal aperture on leaf imprints. Due to difficulties with its reliable measurements, it is not used as a relevant measure of stomatal conductance. We did not estimate stomatal conductance from leaf imprints, instantaneous stomatal conductance (both gsD and gsN) was measured using the LI-600 porometer from Li-cor, we now revised the text to clarify this. It is now clearly indicated in the text.

P9L21: Statistical significance and number of cases taken for the statistical evaluation is not indicated in Figs. 3 and 4. Due to the high and overlapping variation (standard deviation or error?) of both control and heated treatments, I would be very careful to claim that the differences are significant. Figures 3 and 4 are now combined as suggested by other reviewers. The caption in new figure 3 now indicate the number of replicates and state that dotted lines in the boxplots indicate maximum and minimum values, not the standard deviations or errors.

P10L14-16: I would expect that one of the key results, i.e. the decrease in nocturnal stomatal conductance of heated plants, will be presented by a figure. Instead, it is

described in the text without complete statistical evidence. In addition, the averages given here differs from those shown in Table 2. The effect of high night-time temperature on gsN is not the main aim of this manuscript, these results were already published (McAusland et al., 2023) reason why gsN data is only reduced to description in the text and the corresponding citation offered. The paragraph on P10L14-16 doesn't match Table 2 values because the text refers to means across all the experimental germplasm, this is, the 12 genotypes under heat or control, while the information presented on Table 2 refers to a subset of contrasting genotypes.

P10L16: In the methods - section of stomatal conductance measurements - only the adaxial side is mentioned. Here, results on the abaxial side are presented. We have now edited the text in the material and methods section, and we now state that gsN was recorded on both leaf sides, gsD was recorded only in the adaxial side.

P11L6-7: Are these length categories really significantly different? Please, show the necessary details (number of independent measurements, method of testing). Information added to the material and methods section and to the figure.

Referee #1's Review for 24289:

Comments for the Author (Required):
Pinto et al present an interesting study on nighttime wheat physiology, and how it's affected by warming. The study is timely given current concerns on food security under global warming and it is also nicely written. In my opinion, the major problem the paper is facing in its current version is that we are **lacking with a clear set of questions and hypotheses at the end of the introduction**. As a result, the reader loses focus and, while a lot of data is presented, it is hard to know where are the authors heading to given the poor indications given at the introduction.I also think the authors are **over-emphasizing the role of circadian rhythms**. They couldn't measure it at different times during the night and what is really needed **to test that hypothesis is the behaviour right before dawn.** They measure at 9 at 10am and consider the difference as driven by circadian rhythmicity, which is a reasonable assumption, but then we need to know whether environmental conditions were the same (perhaps VPD was higher at 10am, which was driving the decline in gs?).
Below are some specific comments, in case they help improve this paper even more:
We acknowledge the reviewer critiques and as replied to the Editor we have now added a set of hypothesis towards the end of the introduction section. We hope this would help to convey the message of the paper. The environmental conditions are now included in Figure 1, where yes, VPD from 9 to 10 am increase by 0.5Kpa, however, VPD between treatments was the same while gs differ significantly. We have now edited the text to consider the relevance of VPD variation between hours/treatments.

Intro: good intro, but you need a series of specific hypotheses to test. Right now it's written as a descriptive study, so you need to add the patterns you expected to see and the potential processes explaining those. Added. Hypothesis are now clearly stated at the end of the introduction.

P8L4: indicate time of dawn and dusk. Added on Table 1
P8L7: and nighttime? Added on Table 1
P8L3-11: need to have unified convention for gs acronyms. Sometimes there's a dot, sometimes not... my suggestion would be to have subscripts (and the "g" should be in italics). Thanks for the suggestion, the dot is used only for robust averages of gs across phenological stages (gs.B, gs.G) or across the season (gs.Av).
Too many figures. Some could be removed, or moved to the supplement (e.g. Fg.2). Others could be merged (e.g. Figs. 3-4). FIG 3 & 4 are now merged and two figures have been moved to supplemental materials.
Somewhere in the methods, please add the number of reps (or maybe I just missed it). We need more details on measurements of yield and components. The number of reps is indicated however, additional information about the number of measurements was added to the material and methods section and added in Figure 3. The methodology used for yield and components is a conventional utilized elsewhere, in any case we provided references in this section (Pietragalla et al., 2012 and Pask et al., 2013).

Why measure at 9 and 10? They're very close together and not sure the data at 10am is needed. Also, how long did it take to finish one round of measurements? Given that watering occurred every 15 days, there could have been substantial variation in water potentials, and that could have led to differences in values across treatments or cultivars. Do you have data on nocturnal water potentials, or other indicators to show that variation in water availability did not affect the results? What was the effect of heating on VPD? Measurements at 9am were repeated at 10am to have more robust data on gs patterns. And yes, nocturnal heating increased WP in the pre-dawn; these data was presented in a previous work and is properly referenced in the manuscript (i.e. McAusland et al., 2023). As shown in new Figure 1 VPD is not affected by the nighttime heat treatment.

P14L6 - rather than grouping varieties into groups, and doing a t-test, I think it would be more meaningful to do an **ANOVA to look at differences across specific cultivars**. The proposed classification is informal, and a more formal method should be applied, if the authors seek to group the varieties. An ANOVA using Linear Mixed Models was performed and results of genotypic comparison are described in the text. Now, the output of the ANOVA analyses was added to the results section. The two contrasting groups are aimed to quantify the potential gains that can be reached when improving one or more traits, and it is a standard procedure.

P15L3 - increasing VPD often reduces stomatal conductance (although it may increase transpiration), not the other way around. We thank the reviewer for this observation, we now edited text accordingly.

P15L18 - where environmental conditions the same at 9 and 10am? Details of the environmental conditions are now added in the Figure 1., gs at 9 am was measured and again at 10 am in order to have a more robust set of data supporting the trends observed at 9 am.

Referee #2's Review for 24289:

Comments for the Author (Required):

This manuscript describes an interesting evaluation of the stomatal and diurnal effects of stomatal conductance in response to high night temperature. My main concern is that the manuscript describes results from only one study, and that some aspect of the study should be repeated to demonstrate repeatability of the results. Another concern is that the Discussion should better bring together the concepts of time of day, stomatal conductance and night temperature. **Why would plants that were exposed to higher night temperatures be transpiring more in the morning? Is it a residual stress effect from the night time to maintain cooling?**

We thank the reviewer impression of our work, since the first question is indeed the most fundamental lack of knowledge on which we try to contribute with our experiment. We believe that, to some extent, the two questions poised are indeed discussed in the

manuscript (Pages 18-19) and with the overall modifications following the revision the main concepts are better discussed.

Specific comments:

M&M: please provide more details about the growth conditions. What were the dimensions of the flat beds? What were the growth conditions? Were these flat beds placed in the field? Information edited in the material and methods section to provide more details of the growth conditions and Figures 1 and 1S provide additional details.

It would be good to report the relative humidity during the experiment. Added in Figure 1
Nocturnal heating: Please mention the type of heater (fan forced-air heaters/ radiant?) Information added in the material and methods section.
Can the nail polish method really be used to reliably determine stomatal aperture? Is there a reference for this? As replied to the AoB editor´s comments (above) we added key references for this.
Since the heat treatment was initiated at booting stage, please clarify why the authors would expect differences in stomatal density between treatments. No differences on stomatal densities between treatments were expected, reason why Figure 4 presents the averages under heat & control for each of the 12 wheat genotypes.
Table 1: were these heat tolerance categories established according to response to high night temperature or high day temperature? These two categories were established based on previous studies performed at CIMMYT on late sowings. Details added to M&M and to the table with the list of germplasm.
When discussing various concepts, please clarify if the cited references describe wheat or some other species. We thank the comment and tried to be more clear in this topic
The discussion refers to VPD: it would be good to report this for the current study. Added on Figure 1.
The discussion refers to a number of topics that were not explored in this study including respiration and carbohydrates. It would be good to better link the Discussion with the results of the current study.

We thank the reviewer observation, but we believe that there is only one argument that invoke these mechanisms related to respiration and carbohydrate usage and think that this argument goes along the aim of answering the key question of this study while the rest of the discussion centres on the main findings of the study.

Conclusions: the authors should please better tie together the circadian and heat components of the study.

We appreciate the reviewer's suggestion and we have made editions to the second part of our conclusion to tune down the argumentation regarding the circadian rhythms and argue more about the relevance of trade-off stomatal processes associated to nocturnal warming.
